# The improved efficacy of Sifuvirtide compared with enfuvirtide might be related to its selectivity for the rigid biomembrane, as determined through surface plasmon resonance

**DOI:** 10.1371/journal.pone.0171567

**Published:** 2017-02-16

**Authors:** Ping Cao, Guifang Dou, Yuanguo Cheng, Jinjing Che

**Affiliations:** 1 Laboratory of Hematological Pharmacology, State Key Laboratory of Drug Metabolism, Beijing Institute of Transfusion Medicine, Beijing, People's Republic of China; 2 Beijing Institute of Microbiology and Epidemiology, Beijing, People's Republic of China; 3 State Key Laboratory of Toxicology and Medical Countermeasures, Beijing Institute of Pharmacology and Toxicology, Beijing, People's Republic of China; Shanghai Medical College, Fudan University, CHINA

## Abstract

Most mechanistic studies on human immunodeficiency virus (HIV) peptide fusion inhibitors have focused on the interactions between fusion inhibitors and viral envelope proteins. However, the interactions of fusion inhibitors with viral membranes are also essential for the efficacy of these drugs. Here, we utilized surface plasmon resonance (SPR) technology to study the interactions between the HIV fusion inhibitor peptides sifuvirtide and enfuvirtide and biomembrane models. Sifuvirtide presented selectivity toward biomembrane models composed of saturated dipalmitoylphosphatidylcholine (DPPC) (32-fold higher compared with unsaturated 1-palmitoyl-2-oleoyl-sn-glycero-3-phosphocholine [POPC]) and sphingomyelin (SM) (31-fold higher compared with POPC), which are rigid compositions enriched in the HIV viral membrane. In contrast, enfuvirtide showed no significant selectively toward these rigid membrane models. Furthermore, the bindings of sifuvirtide and enfuvirtide to SM bilayers were markedly higher than those to monolayers (14-fold and 23-fold, respectively), indicating that the inner leaflet influences the binding of these drugs to SM bilayers. No obvious differences were noted in the bindings of either peptide to the other mono- and bilayer models tested, illustrating that both peptides interact with these membranes through surface-binding. The bindings of the inhibitor peptides to biomembranes were found to be driven predominantly by hydrophobic interactions rather than electrostatic interactions, as determined by comparing their affinities to those of positively charged 1-palmitoyl-2-oleoyl-sn-glycero-3-ethylphosphocholine (EPC) to zwitterionic membrane models. The improved efficiency of sifuvirtide relative to enfuvirtide might be related to its ability to adsorb on rigid lipidic areas, such as the viral envelope and lipid rafts, which results in an increased sifuvirtide concentration at the fusion site.

## Introduction

The processes of the binding of human immunodeficiency virus type 1 (HIV-1) to its target cell and membrane fusion depend on the viral envelope glycoproteins gp41 and gp120 [1]. Synthetic peptides based on gp41 heptad repeat (HR) 2 are being developed to target the viral HR1/HR2 interaction [2]. Enfuvirtide (T-20, Fuzeon®) is currently the only HIV-1 fusion inhibitor peptide that is clinically approved by the U.S. Food and Drug Administration (FDA) [3,4]. Nonetheless, strains that exhibit resistance to this peptide have emerged [5]. Sifuvirtide is a potential fusion inhibitor that was developed by FusoGen Pharmaceuticals, Inc., and its antiviral activity has been shown through *in vitro* experiments. A cell-cell fusion assay revealed that the effective concentration for achieving 50% inhibition (IC50) of sifuvirtide is 1.2 ± 0.2 nm, whereas that of enfuvirtide is 23 ± 6 nm [6]. The analysis of cell-mediated viral infections demonstrated that sifuvirtide presents markedly higher potency than enfuvirtide against a wide range of primary and laboratory-adapted HIV-1 strains and enfuvirtide-resistant HIV-1 strains [7,8]. We previously reported the results of a pharmacokinetic assessments of two clinical studies of sifuvirtide in Chinese HIV patients [9], which showed that the efficacy of a once-daily administration of 20 mg of sifuvirtide is equivalent to that of a twice-daily administration of 90 mg of enfuvirtide. Sifuvirtide also has a markedly longer T_1/2_ (39 h) than enfuvirtide (3.8 h), which implies that sifuvirtide has improved clinical pharmacokinetic characteristics and demonstrates that this drug is a suitable and promising alternative.

The development of new fusion inhibitors has focused on improving their affinity to the HR1 region of gp41 [10–12]. However, the interactions of fusion inhibitors with biomembranes are also important for determining their mode of action and activity because the inhibition process must occur in extreme confinement between both the viral and the cellular membranes [13]. For instance, enfuvirtide [1,14] and sifuvirtide [2,15] interact with lipid membranes. Sifuvirtide might adsorb onto the surface of rigid membranes [15], and membranes can thus serve as catalysts [16] of the inhibition process by providing an increased concentration of peptides near the fusion site. Nonetheless, the mode of action and the dependence on other rigid lipids, such as sphingomyelin (SM), which is characteristic of lipid rafts and viral envelopes [17–20], remain incompletely understood.

Over the last few decades, surface plasmon resonance (SPR) technology using a BIAcore biosensor has been shown to be a powerful tool for investigating the binding behavior of macromolecules [21]. The most obvious advantages of SPR over other techniques are the following: the direct and rapid determination of the kinetics of the binding process, the fact that sample labelling is not required, and the small amounts of sample that need to be used in the assay [22]. In biochemistry, SPR is used primarily to study protein-protein and protein-DNA interactions [2], and this technique has also been used to study protein/peptide-membrane interactions [23], although the methods for this analysis are not as well developed. The commercialization of sensor chips dedicated to lipid systems (i.e., the hydrophobic association [HPA] chip and the lipid-capture [L1] chip) has enabled the easy study of protein/peptide-membrane interactions through the manipulation of the lipid composition of the immobilized membrane [22]. Furthermore, it is possible to differentiate between surface adsorption and insertion into the hydrophobic core of the membrane through the use of both HPA and L1 chips. Indeed, if the peptide binds only to the interface, similar equilibrium constants should be observed with both chips [24].

The aim of this work was to study the interactions of sifuvirtide and enfuvirtide with rigid membrane models using the SPR technique, and the ultimate goal was to clarify the specific molecular mode of HIV fusion inhibitor binding at the membrane level and demonstrate the importance of membrane interactions in improving the efficiency of new HIV fusion inhibitors.

## Materials and methods

### Materials

Sifuvirtide (SWETWEREIENYTRQIYRILEESQEQQDRNERDLLE, MW 4727) and enfuvirtide (YTSLIHSLIEESQNQQEKNEQELLELDKWASLWNWF, MW 4492) were synthesized by GL Biochem, Ltd. (Shanghai, China). 1-Palmitoyl-2-oleyl-sn-glycero-3-phosphocholine (POPC), 1,2-dipalmitoyl-sn-glycero-3-phosphocholine (DPPC), 1-palmitoyl-2-oleoyl-sn-glycero-3-ethylphosphocholine (EPC) and SM were purchased from Avanti Polar-Lipids (Alabaster, AL, USA). HBS-N buffer (4-(2-hydroxyethyl)-1-piperazineethanesulfonic acid [HEPES] + NaCl), 0.2-M NaOH and the Biacore Maintenance Kit were purchased from General Electric (CT, USA). N-octyl β-D-glucopyranoside and bovine serum albumin (BSA) were purchased from Sigma-Aldrich.

### Preparation of Small Unilamellar Vesicles (SUVs)

SUVs comprising various components were prepared in HBS-N buffer. Briefly, dry lipids were separately dissolved in chloroform, and the solvents were evaporated using a rotary evaporator. The lipids were then resuspended in HBS-N buffer at a concentration of 0.5 mM with respect to the phospholipids. The resultant lipid suspensions were passed through a liposome extruder containing a 50-nm polycarbonate filter 19 times until a clear solution was obtained. The sizes of the SUVs obtained were measured through dynamic light scattering (DLS).

### Preparation of vesicle-coated sensor chips

Biosensor experiments were conducted with a BIAcore T200 (GE) instrument using HPA and L1 sensor chips. The HPA chip is composed of aliphatic chains covalently bound to a gold surface, and a hybrid lipid monolayer is formed when the chip comes in contact with vesicles. The L1 chip contains hydrophobic aliphatic chains with exposed polar headgroups; thus, when the chip comes in contact with vesicles, a lipid bilayer forms [24]. We followed the protocol described by Papo and Shai [24].

Briefly, SUVs (80 μL, 0.5 mM) were applied to the HPA (or L1) chip surface at a low flow rate of 2 μL/min. To remove any multilamellar structures from the lipid surface, NaOH (25 μL, 10 mM) was injected at a flow rate of 50 μL/min. BSA was then injected (10 μL, 0.1 mg/mL) to confirm complete coverage of the nonspecific binding sites. The monolayer (or bilayer, in the case of the L1 chip) linked to the chip surface was then used as a model membrane surface for studying peptide-membrane binding.

### Binding analysis using the SPR biosensor

Peptide solutions were prepared by dissolving sifuvirtide and enfuvirtide in HBS-N buffer at concentrations ranging from 1.95 to 62.5 μM. The peptide solutions were injected over the lipid surface at a flow rate of 5 μL/min and then replaced by HBS-N buffer to allow peptide dissociation for 1200 s. The lipid monolayer (or bilayer) was completely removed through the injection of 40-mM N-octyl β-D-glucopyranoside, and each peptide injection was performed on a freshly generated lipid surface. All binding experiments were performed at 25℃. A sensorgram was obtained by plotting the response over time.

### Analysis of the SPR data

All of the data were evaluated using BIAevaluation software (GE). The sensorgrams were globally fit to a steady-state affinity model: [peptide + vesicle] ↔ peptide + vesicle. The rate constants for the dissociation (k_d_) of the peptide from phospholipid surfaces were determined by fitting the dissociation kinetics data to the following equation describing a single-phase dissociation process:

$$\text{dR}/\text{dt}={\text{-k}}_{\text{d}}∙\text{R}$$ (1)

The rate constants for association (k_a_) were determined from individual association kinetics data using the following equation:

$$\text{R}=\text{C}∙{\text{k}}_{\text{a}}∙{\text{R}}_{\text{max}}∙(1{\text{-e}}^{\text{-}(\text{C}∙\text{ka}+\text{kd})∙\text{t}})/(\text{C}∙{\text{k}}_{\text{a}}+{\text{k}}_{\text{d}})$$ (2)

where R_max_ is the maximal binding capacity of the immobilized ligand surface expressed in RU and C is the concentration of the peptide in solution. The values of the equilibrium dissociation constants (K_D_) were calculated as k_d_/k_a_. Because K_D_, which has the dimensions of concentration, equals the concentration of free peptide at which half of the total molecules of phospholipids are associated with the peptide, additional lines parallel to the y-axis were added to the figures to mark the location of the K_D_ value.

## Results

### Preparation of SUVs and monolayer/bilayer vesicle-coated sensor chips

SUVs with different rigid compositions and electric charges were prepared by extrusion through polycarbonate filters. The average diameters of the SUVs were mainly 80–90 nm, as measured through DLS (Table 1).

**Table 1:** Average Hydrodynamic Diameter of the SUVs Particles Measured Using DLS technique.

Lipid	Average Diameter ± SD (nm)	PdI ± SD
POPC	90.15±0.6830	0.080±0.026
POPC:DPPC(1:1)	84.85±0.4518	0.028±0.009
POPC:DPPC(1:2)	83.88±0.7100	0.083±0.017
DPPC	86.13±0.1801	0.042±0.028
EPC	79.48±0.3408	0.057±0.026
SM	83.49±0.4652	0.054±0.038

POPC/POPC:DPPC(1:1)/POPC:DPPC(1:2)/DPPC/EPC/SM monolayers and bilayers were absorbed onto the HPA and L1 chips, respectively. The HPA and L1 chips were regenerated with 40-mM N-octyl β-D-glucopyranoside after each experiment, and the drift in the signal was less than 10 RU relative to the baseline before the experiment, indicating that the system was stable.

### Binding affinity of sifuvirtide to lipid monolayers and bilayers

**Fig 1:**
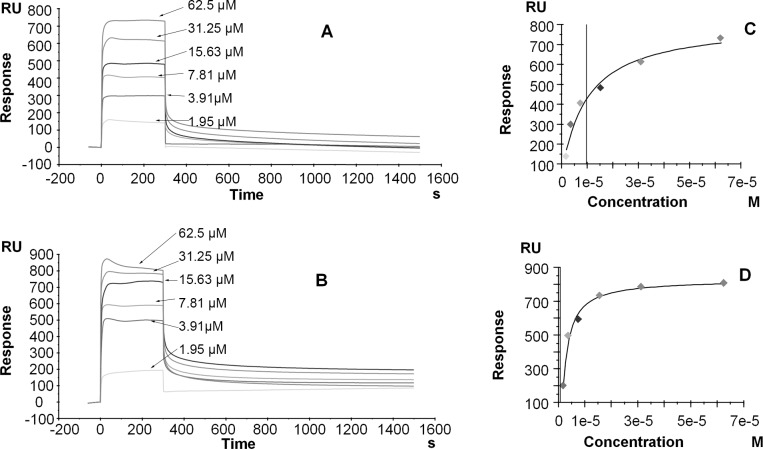
Bindings of Sifuvirtide to SM Monolayers (HPA Chip) and Bilayers (L1 Chip). Panels A and B: Sensorgrams of the binding of sifuvirtide to an SM monolayer (panel A) and bilayer (panel B). Panels C and D: Corresponding relationships between the equilibrium binding response (RUeq) and the peptide concentration. The data were fit using the steady-state affinity model. The sifuvirtide concentrations used were 1.95, 3.91, 7.81, 15.63, 31.25, and 62.5 μM. The additional lines parallel to the y-axis indicate the K_D_ value.

The sensorgrams of the bindings of sifuvirtide to SM monolayers (HPA chip) and bilayers (L1 chip) are shown with typical representative tracings in panels A and B in Fig 1. The binding of sifuvirtide to SM bilayers showed lower dissociation than that to SM monolayers, and the response levels of the binding (except at 1.95 μM) to the SM bilayer (500–800 RU) were slightly higher than those of the binding to the SM monolayer (300–700 RU), demonstrating that the inner layer of the membrane might influence the interaction of sifuvirtide with the SM bilayer. The sensorgrams of other lipid compositions (POPC/POPC:DPPC(1:1)/POPC:DPPC(1:2)/DPPC/EPC) did not reveal obvious differences between monolayers and bilayers, indicating that sifuvirtide interacted with these membranes via surface binding (S1 and S2 Figs).

Our system reached binding equilibrium during the injection of the sample (Fig 1, panels C and D); therefore, the equilibrium dissociation constants could be calculated using a steady-state affinity model. Table 2 shows the dissociation constants obtained for sifuvirtide and the ratio of the bilayer-to-monolayer affinities. The results showed that the dissociation constants of sifuvirtide from monolayers and bilayers of POPC, POPC:DPPC(1:1), POPC:DPPC(1:2) and DPPC decreased as the saturation of the phospholipids increased, illustrating that the affinity of sifuvirtide increased as its saturation level increased. The dissociation constants for sifuvirtide from EPC monolayers and bilayers were similar to those measured for the POPC:DPPC(1:1 and 1:2) mono- and bilayers, indicating that electrostatic interactions were not more important than hydrophobic interactions in the membrane-binding behavior of sifuvirtide. The binding of sifuvirtide to the lipid monolayers mentioned above was similar to its binding to bilayers, indicating that the interactions were not influenced by the inner layers of the membranes. Thus, sifuvirtide interacted with these membranes by surface binding without inserting into the bilayers or forming pores. However, its binding to SM bilayers was 14-fold higher than that to monolayers, indicating that the inner layer exerts an influence. Thus, sifuvirtide might be partially inserted into the outer leaflet of SM bilayers. This result agrees with the results presented in panels A and B in Fig 1. The ratios of the dissociation constants of sifuvirtide with POPC and other lipid compositions are listed in Table 3. The affinities of sifuvirtide to monolayers and bilayers of DPPC were 25-fold and 32-fold higher than those for POPC, and this finding is similar to those obtained previously using other methods [2]. These results demonstrate that sifuvirtide has greater affinities to phospholipids with higher hydrocarbon saturation levels. The affinity of sifuvirtide for SM bilayers was 31-fold higher than that for POPC, revealing that sifuvirtide has a significantly higher affinity for SM bilayers. Because DPPC and SM are rigid components of biomembranes, we can deduce that sifuvirtide binds to rigid membranes in a selective manner.

**Table 2:** Equilibrium Dissociation Constants Determined by SPR for the Interaction of Sifuvirtide with Lipid Monolayers (HPA Chip) and Bilayers (L1 Chip)*.

Lipid	K_D_±SE^**^(×10^−6^ M)		K_D monolayer_/K_D bilayer_
	Monolayer	Bilayer	
POPC	78.7±59	21.4±6.5	3.7
POPC:DPPC(1:1)	10.9±9.6	5.10±3.0	2.1
POPC:DPPC(1:2)	8.22±4.4	4.42±3.1	1.9
DPPC	3.18±2.1	0.663±0.58	4.8
EPC	12.8±8.1	4.72±2.9	2.7
SM	9.81±4.3	0.690±0.61	14

*Calculated by Derived According to a Steady-State Affinity Model.

**Standard Error.

**Table 3:** The Ratio of Equilibrium Dissociation Constants.

Lipids	Sifuvirtide		Enfuvirtide	
	Monolayer	Bilayer	Monolayer	Bilayer
K_D POPC_/K_D POPC:DPPC(1:1)_	7.2	4.2	4.2	2.6
K_D POPC_ /K_D POPC:DPPC(1:2)_	9.6	4.8	1.8	2.6
K_D POPC_ /K_D DPPC_	25	32	1.0	2.9
K_D POPC_ /K_D EPC_	6.2	4.5	0.50	0.26
K_D POPC_ /K_D SM_	8.0	31	0.036	1.6

### Binding affinity of enfuvirtide for lipid monolayers and bilayers

**Fig 2:**
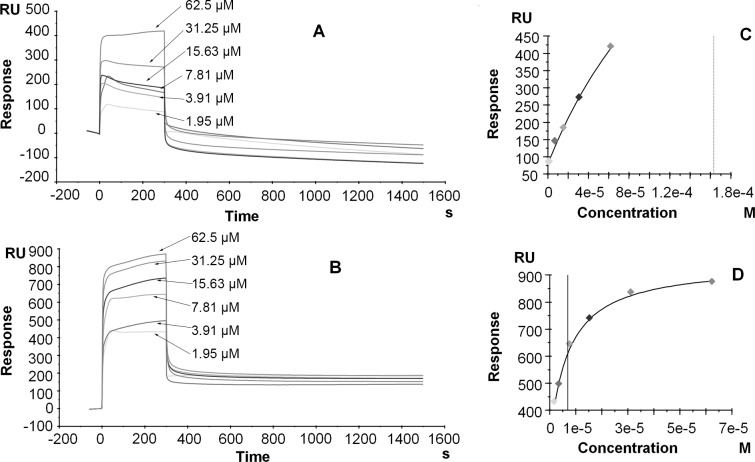
Bindings of Enfuvirtide to SM Monolayers (HPA Chip) and Bilayers (L1 Chip). Panels A and B: Sensorgrams of the binding of enfuvirtide to an SM monolayer (panel A) and bilayer (panel B). Panels C and D: Corresponding relationships between the equilibrium binding response (RUeq) and the peptide concentration (C). The data were fit using the steady-state affinity model. The enfuvirtide concentrations used were 1.95, 3.91, 7.81, 15.63, 31.25, and 62.5 μM. The additional lines parallel to the y-axis indicate the K_D_ value.

The sensorgrams of the binding of enfuvirtide to SM monolayers and bilayers are shown in panels A and B in Fig 2. The maximum response of SM bilayers (~900 RU) was more than two-fold higher than that of monolayers (~400 RU). Moreover, the response of SM monolayers decreased immediately after the injection of enfuvirtide, suggesting rapid dissociation. In contrast, a certain level of response from SM bilayers was maintained after the injection of enfuvirtide, and this was followed by a slower dissociation process. These results indicate that the membrane’s inner layer increased the binding of enfuvirtide to the SM bilayer. The sensorgrams obtained with other lipid compositions (POPC, POPC:DPPC(1:1 and 1:2), DPPC, and EPC) did not show obvious differences between monolayers and bilayers, demonstrating that enfuvirtide interacted with these membranes by surface binding (S3 and S4 Figs).

The equilibrium dissociation constants of enfuvirtide calculated using a steady-state affinity model (Fig 2, panels C and D), as well as the ratios of the bilayer-to-monolayer affinities, are shown in Table 4. Enfuvirtide exhibited similar dissociation constants from both monolayers and bilayers of phospholipids of various compositions (POPC, POPC:DPPC(1:1 and 1:2), and DPPC), demonstrating no selectivity for the hydrocarbon saturation level. The dissociation constants of enfuvirtide from positively charged EPC monolayers (1.16×10^−5^ M) and bilayers (4.30×10^−5^ M) were slightly higher than those from zwitterionic membranes, such as POPC, POPC:DPPC(1:1 and 1:2), and DPPC, suggesting that enfuvirtide has a slightly weaker affinity for EPC than for zwitterionic membranes. Therefore, hydrophobic interactions play a relatively important role in the binding of enfuvirtide to biomembranes. The dissociation constant of enfuvirtide from SM monolayers (1.63×10^−4^ M) was markedly higher than those from other zwitterionic membranes, suggesting that enfuvirtide has very weak affinity for SM monolayers. The bindings of enfuvirtide to monolayers of POPC, POPC:DPPC(1:1 and 1:2), DPPC and EPC were similar to those to bilayers, indicating that the interactions are not influenced by the membranes’ inner layers. Thus, enfuvirtide interacts with these membranes by surface binding. In contrast, its binding to SM bilayers was 23-fold higher than that to monolayers, suggesting a dependence on the bilayer structure and that enfuvirtide might be slightly inserted into the SM bilayer. This result is in agreement with the results presented in panels A and B in Fig 2 and is similar to the results obtained for sifuvirtide. The results presented on Table 3 indicate that no significant difference exists between the affinities of enfuvirtide to POPC, POPC:DPPC (1:1), POPC:DPPC (1:2) and DPPC monolayers and bilayers and that enfuvirtide has substantially weaker affinity to SM monolayers, which suggests that this compound shows no selectivity for rigid phospholipids, a result that differs from that obtained for sifuvirtide.

**Table 4:** Equilibrium Dissociation Constants Determined by SPR for the Interaction of Enfuvirtide from Monolayers (HPA Chip) and Bilayers (L1 Chip)*.

Lipid	K_D_±SE^**^(×10^−6^ M)		K_D monolayer_/K_D bilayer_
	Monolayer	Bilayer	
POPC	5.83±1.1	11.3±1.9	0.52
POPC:DPPC(1:1)	1.39±1.1	4.41±1.8	0.32
POPC:DPPC(1:2)	3.28±3.0	4.34±1.8	0.75
DPPC	5.69±3.9	3.93±2.6	1. 5
EPC	11.6±2.1	43.0±9.0	0.27
SM	163±78	6.98±1.6	23

*Calculated by Derived According to a Steady-State Affinity Model.

**Standard Error.

## Discussion

In this study, the unsaturated phospholipid POPC was selected to mimic the ordinary eukaryotic plasma membrane. In HIV membranes, the SM and saturated phosphocholine (PC) levels are 3.2-fold and 3.6-fold higher, respectively, compared with those of eukaryotic plasma membranes [17]. Therefore, phospholipids containing the saturated phospholipids DPPC and SM were selected as the HIV viral membrane model. Monolayer and bilayer models were used to study the selectivity of HIV fusion inhibitors for rigid components and the modes of these interactions.

### Mode of interactions between HIV fusion inhibitors and biomembranes

It has been reported that lipid membranes play an important role in the modes of action of sifuvirtide and other HIV fusion inhibitors, such as enfuvirtide and T-1249 [1,14,15]. According to our results, the affinity of sifuvirtide for DPPC bilayers is 32-fold higher than that for POPC bilayers, suggesting its capability to bind to saturated phospholipids in a selective manner. This result agrees with those obtained using fluorescence spectroscopy techniques [2]. However, enfuvirtide presented similar affinities for phospholipids membranes with various levels of hydrocarbon saturation, suggesting that it exhibits no selectivity for saturated phospholipids, as determined through partition experiments and fluorescence resonance energy transfer analysis [1,25]. Instead, these interactions might differ depending on the structural properties of the peptides. Compared with the sequence of enfuvirtide, sifuvirtide has its deep pocket-binding domain (PBD) in the N terminus, which is believed to target the hydrophobic pocket of HR1, but lacks the tryptophan-rich domain (TRD), which is also known as the lipid-binding domain (LBD), in the C terminus [26]. Aromatic residues, particularly Trp, have been reported play a key role in the high affinity of proteins for PC membranes [26–28]. Compared with the dissociation constants (K_D_) of sifuvirtide from POPC (7.87×10^−5^ M for monolayers and 2.14×10^−5^ M for bilayers, Table 2), enfuvirtide was found to interact more strongly with POPC vesicles (5.83×10^−6^ M for monolayers and 1.13×10^−5^ M for bilayers, Table 4), possibly due to the presence of the Trp-rich region in its C terminus [2]. The PBD has been reported to be essential for improving the anti-HIV activity [29–31]. Because the PBD of sifuvirtide might be related to its selectivity for saturated phospholipids, the binding properties of T-1249, another fusion inhibitor peptide with both a TRD and a PBD, were evaluated, and the results showed that the affinities of T-1249 to POPC:DPPC (1:1) (K_D_ = 1.24×10^−6^ M) and DPPC (K_D_ = 3.95×10^−6^ M) were 9.6-fold and 3-fold higher than those of the peptide to POPC (K_D_ = 1.19×10^−5^ M), indicating that peptides with both a TRD and PBD have higher affinities to biomembranes composed of both POPC and DPPC. The affinity of sifuvirtide for SM bilayers increased significantly (31-fold relative to that for POPC, Table 3), indicating that sifuvirtide binds to rigid SM bilayers in a selective manner. In contrast, enfuvirtide was found to present similar affinity for SM bilayers and other zwitterionic bilayers (Table 3), which suggests that this peptide exhibits no selectivity for SM. These results illustrate that sifuvirtide binds to rigid biomembrane models selectively, whereas enfuvirtide does not.

Both sifuvirtide and enfuvirtide are negatively charged peptides and can bind to the positively charged EPC via electrostatic interactions. The dissociation constants obtained for sifuvirtide (4.72×10^−6^ M, Table 2) and enfuvirtide (4.3×10^−5^ M, Table 4) from EPC bilayers were higher than those obtained for DPPC (6.63×10^−7^ M and 3.93×10^−6^ M, respectively), demonstrating that the interactions of both peptides with membranes are driven predominantly by hydrophobic interactions.

Papo and Shai [24] studied the modes of action of membrane-active peptides by comparing the affinities of the peptides for monolayers and bilayers. The advantage of comparing both monolayers and bilayers is that the effect of the membrane’s inner layer could be investigated directly with regard to the peptides’ binding properties. If a peptide inserts into the hydrophobic core of the membrane, the sensorgrams of the binding between the peptide and the lipid monolayers should show markedly lower response levels compared with those recorded for bilayers. The affinity of sifuvirtide for SM bilayers was 14-fold higher than that for monolayers. Although enfuvirtide was found to exhibit no selectivity for SM, its binding to SM bilayers was 23-fold higher than that to monolayers. These results indicate that the membrane’s inner layer increased the binding of the peptides to the SM bilayer. A pore-forming toxin, equinatoxin II, has been reported to preferentially bind to SM-containing membranes and create pores [28], demonstrating an important role of the inner layer of SM bilayers in interactions between proteins/peptides and SM. The binding of both sifuvirtide and enfuvirtide to other lipid monolayers showed similar response levels to those obtained using bilayers, which suggests that this binding was not influenced by the inner layer; in other words, both peptides bound to the surfaces of these membranes. Consistent with our results, Franquelim et al. [2] showed that sifuvirtide did not noticeably affect the lipid bilayer structure.

### Relationship between the characteristics of the bindings of the fusion inhibitors to biomembranes and their clinical efficacies

The improved clinical efficiency of sifuvirtide relative to that of enfuvirtide [9] might be related to its ability to adsorb on rigid lipidic areas of the viral envelope and cell membrane, where most of the fusion-related glycoproteins and receptors are inserted [17]. Sargent et al. [32] proposed that surface accumulation is a very effective method for enhancing the receptor binding of ligand molecules; this effect is termed “membrane catalysis”. According to this theory, lipid bilayers might enhance the fusion process (sometimes referred to as catalyst-like activity [16]) by concentrating the inhibitor peptides near the fusion sites [2,14]. This process is in line with the conclusions obtained by Franquelim et al. [2]. Thus, membranes rich in rigid lipids, such as DPPC and SM, might be supplementary targets of HIV fusion inhibitors. Sifuvirtide was found to have a markedly longer T_1/2_ (39 h) than enfuvirtide (3.8 h) [9], possibly because the accumulation of the peptide near the fusion site led to slower clearance (CL). The lipid specificity of HIV fusion inhibitors was described in this work using biomembrane models with various lipid compositions; however, the natural biomembranes of viruses and cells cannot be simulated completely by artificial membranes. In this case, vesicular preparations of lipids isolated from cells or viruses might be useful [22].

In summary, through the application of SPR to study the interactions of peptide HIV fusion inhibitors with biomembrane models, we clarified the specific molecular modes of action of the peptide HIV fusion inhibitors sifuvirtide and enfuvirtide at the membrane level. The importance of membrane rigidity in the mode of action of sifuvirtide was demonstrated, indicating that membrane rigidity might contribute to the improved efficacy of this compound relative to that of enfuvirtide.

## Supporting information

S1 Fig Bindings of Sifuvirtide to Monolayers (HPA Chip). (PDF)

S2 Fig Bindings of Sifuvirtide to Bilayers (L1 Chip). (PDF)

S3 Fig Bindings of Enfuvirtide to Monolayers (HPA Chip). (PDF)

S4 Fig Bindings of Enfuvirtide to Bilayers (L1 Chip). (PDF)
