# Supplementary figures and images for: The improved efficacy of Sifuvirtide compared with enfuvirtide might be related to its selectivity for the rigid biomembrane, as determined through surface plasmon resonance
Source: PLoS One. 2017 Feb 16;12(2):e0171567. doi: 10.1371/journal.pone.0171567 (PMC5312942; doi:10.1371/journal.pone.0171567)

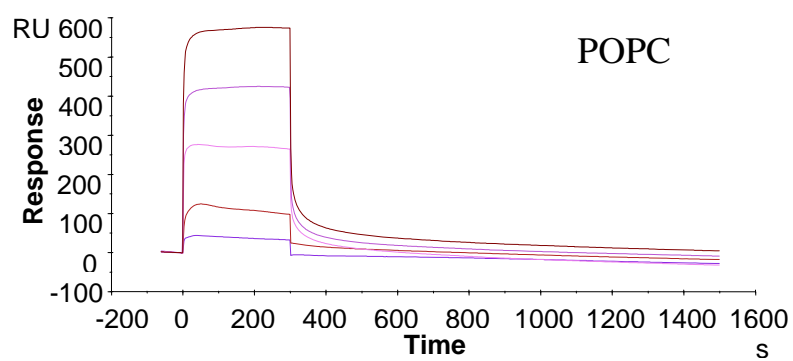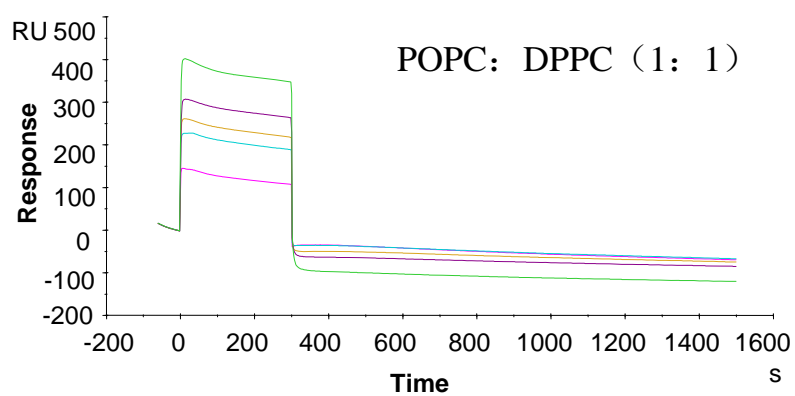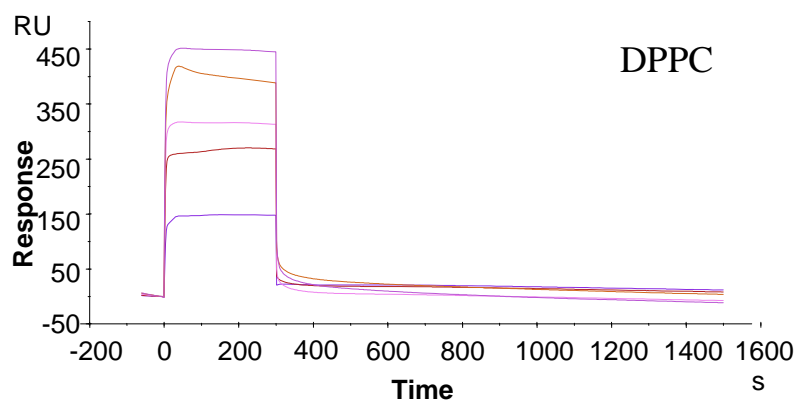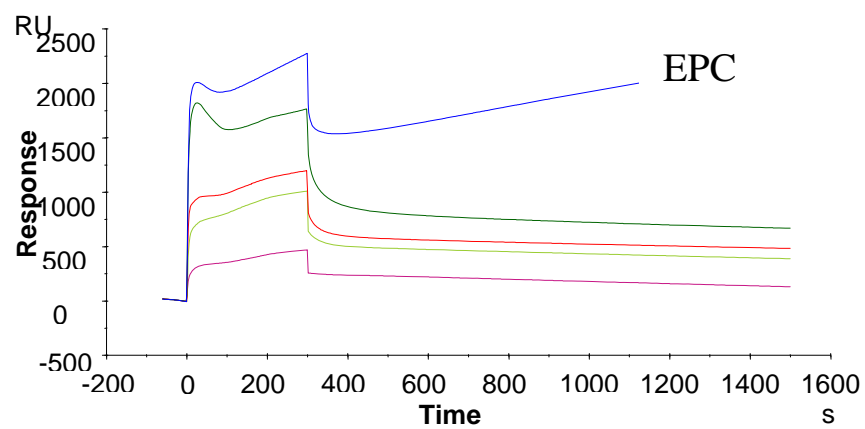

Supplement: S1 Fig — (PDF) [file pone.0171567.s001.pdf]

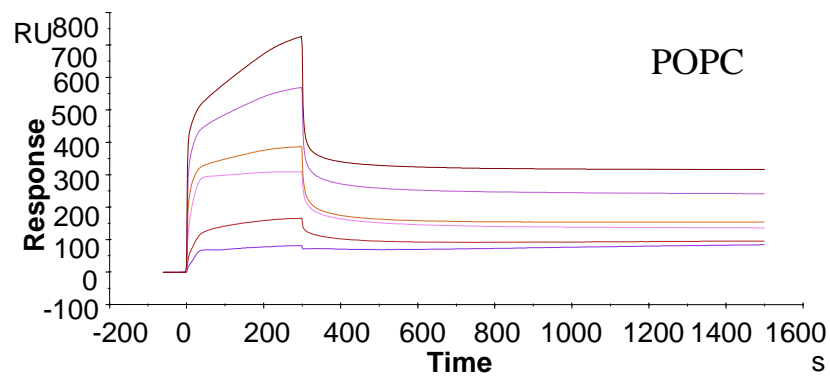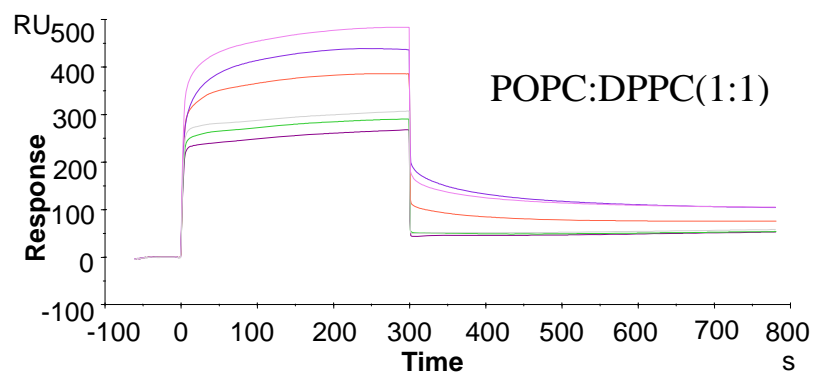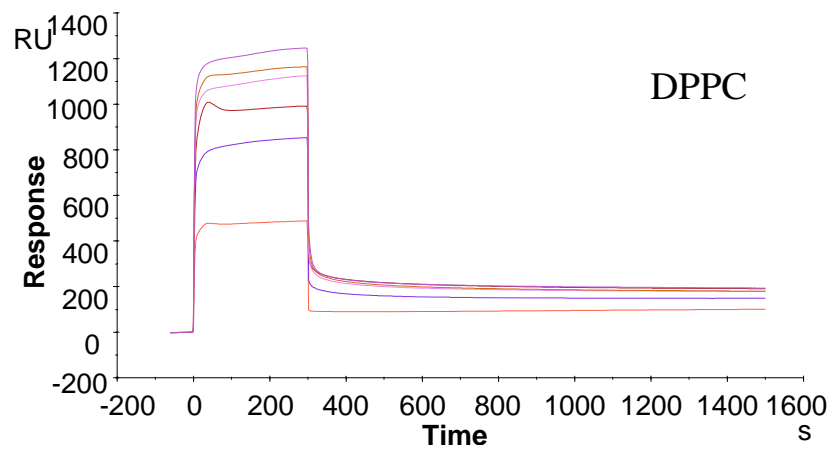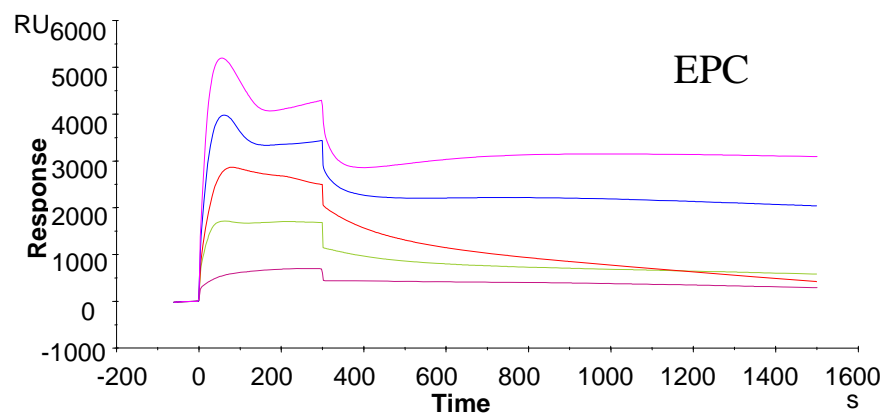

Supplement: S2 Fig — (PDF) [file pone.0171567.s002.pdf]

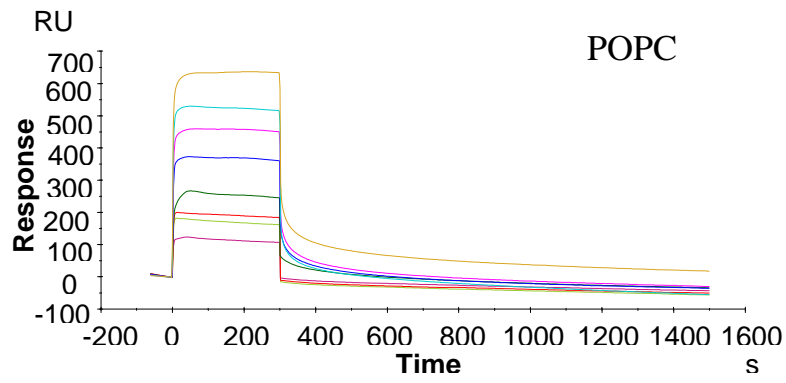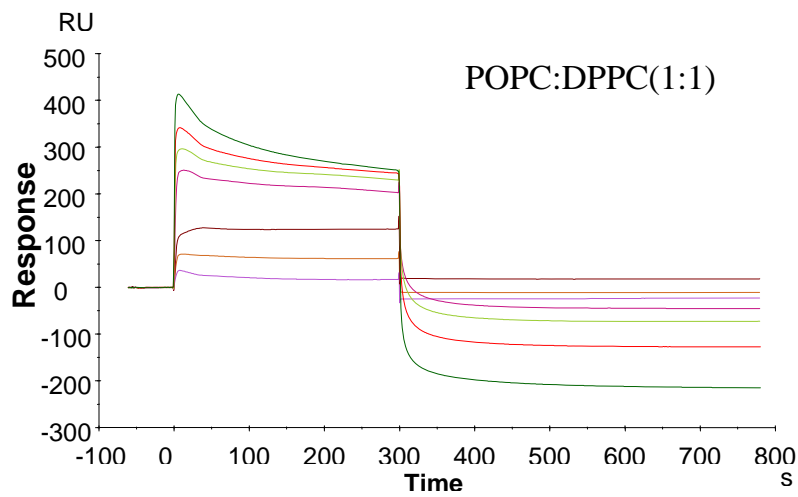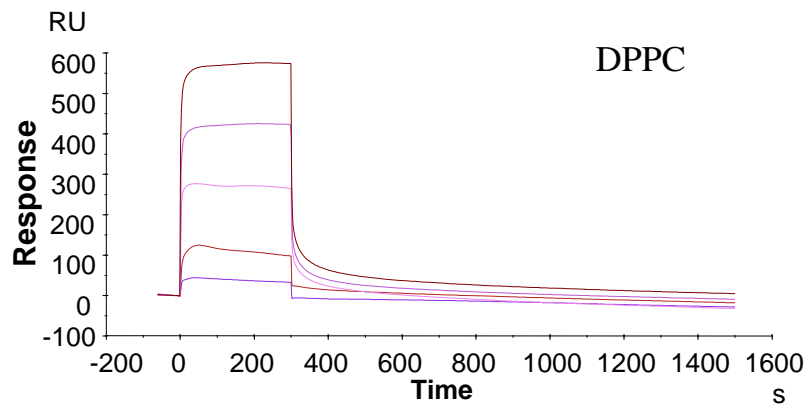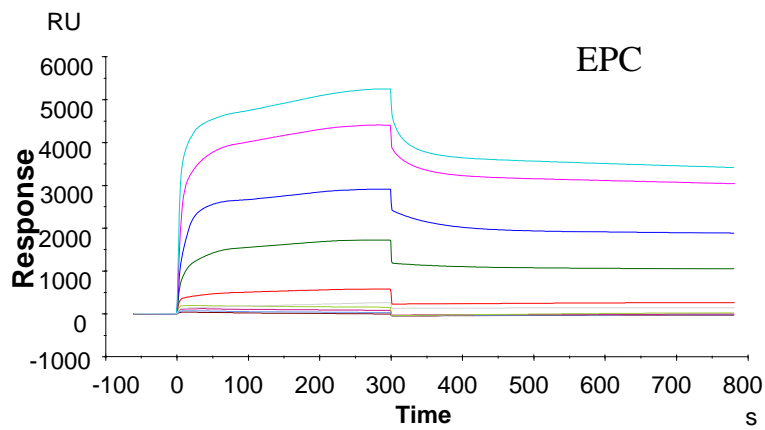

Supplement: S3 Fig — (PDF) [file pone.0171567.s003.pdf]

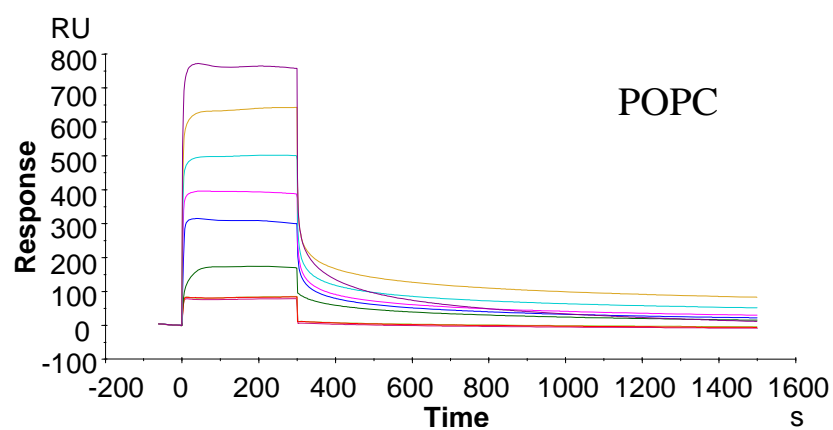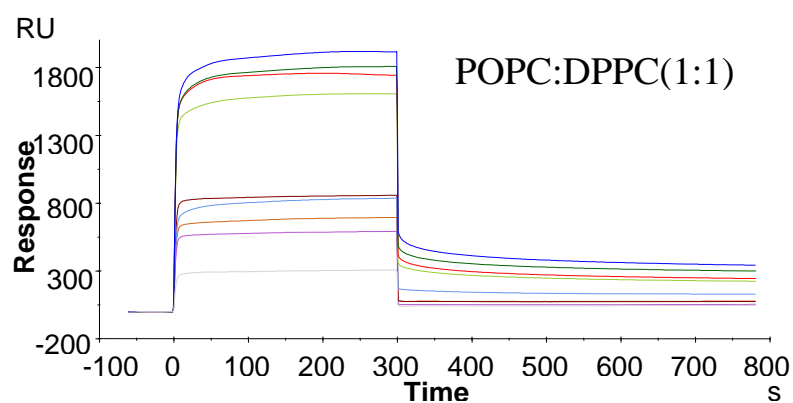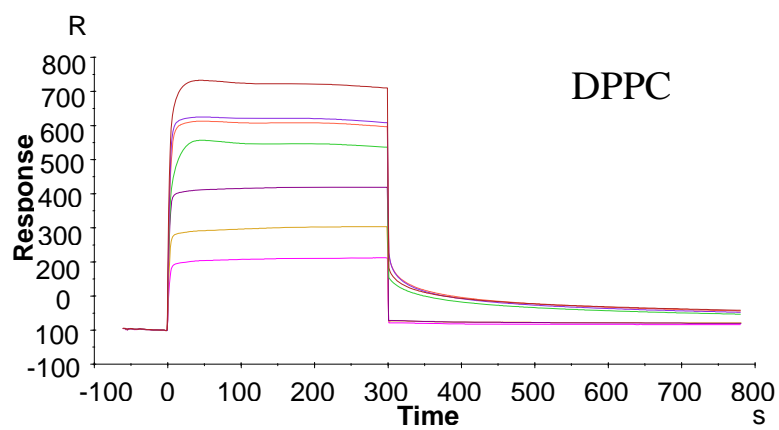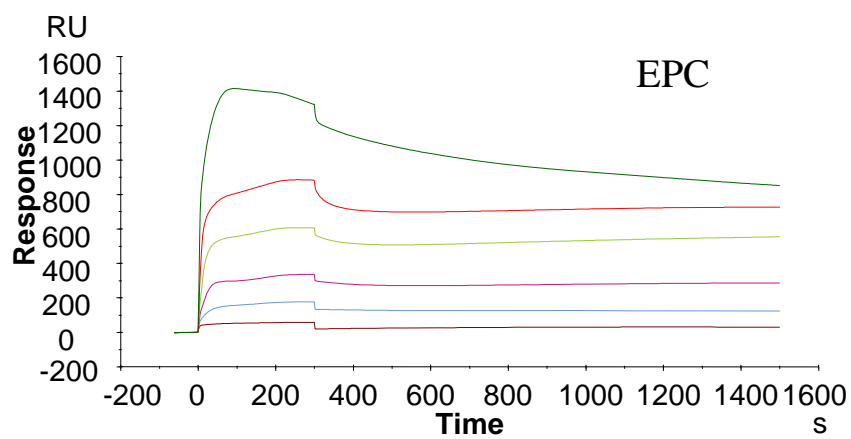

Supplement: S4 Fig — (PDF) [file pone.0171567.s004.pdf]
